# Obesity and cardiovascular risk factors among internists in Indonesia

**DOI:** 10.3389/fendo.2025.1594678

**Published:** 2025-10-01

**Authors:** Sally Aman Nasution, Lugyanti Sukrisman, Eka Ginanjar, Evy Yunihastuti, Simon Salim, Rudy Hidayat, Muhadi Muhadi, Siprianus Ugroseno Yudho Bintoro, Asri Ludin Tambunan, Hery Djagat Purnomo, Andi Makbul Aman, Mohammad Robikhul Ikhsan, Ahmad Mekah, Angkasa R. Hamdan, Indra Wijaya, I. Made Siswadi Semadi, Wira Gotera, Putri Muthia, Zen Ahmad, Muhammad Diah, Nur Samsu, Santy A. P. Perdhana, Tatar Sumandjar, Johana Prihatini, Juwanto Wakimin, Linda Wilhelma Ancella Rotty, Harnavi Harun, Kuntjoro Yakti, Erwin Erwin, Lukman Pura, Abimanyu Abimanyu, Sutiadi Kusuma, Suharno Hakim, Riskadwi Septianti, Budiman Gunawan, Faradiesa Addiena, Kongko H. Nursetiyanto, Arif Koswandi, Andreas N. F. Lewai, Joko Anggoro, Maria Nainggolan, Arfan Sanusi, Petrus Irianto, Komariatun Komariatun, Annelin Kurniati, Haeril Aswar, Nelyan H. Mokoginta, Leily D. Pawa, Edwin Ambar, Feliks Duwit

**Affiliations:** ^1^ Faculty of Medicine Universitas Indonesia, Cipto Mangunkusumo Hospital, Jakarta, Special Capital Region of Jakarta, Indonesia; ^2^ Faculty of Medicine Universitas Airlangga, Dr. Soetomo General Academic Hospital, Surabaya, East Java, Indonesia; ^3^ Department of Internal Medicine Drs. H. Amri Tambunan General Hospital, Deli Serdang, North Sumatra, Indonesia; ^4^ Faculty of Medicine Universitas Diponegoro, Dr. Kariadi Hospital, Semarang, Central Java, Indonesia; ^5^ Department of Internal Medicine Ibnu Sina Yayasan Wakaf Universitas Muslim Indonesia Hospital, Makassar, South Sulawesi, Indonesia; ^6^ Faculty of Medicine, Public Health, and Nursing Universitas Gadjah Mada, Dr. Sardjito Hospital, Yogyakarta, Special Region of Yogyakarta, Indonesia; ^7^ Department of Internal Medicine, Sari Asih Sangiang Hospital, Tangerang, Banten, Indonesia; ^8^ Faculty of Medicine Universitas Padjadjaran, Hasan Sadikin Hospital, Bandung, West Java, Indonesia; ^9^ Faculty of Medicine Universitas Udayana, Prof. dr. I Goesti Ngoerah Gde Ngoerah General Hospital, Denpasar, Bali, Indonesia; ^10^ Faculty of Medicine Universitas Sriwijaya, Mohammad Hoesin General Hospital, Palembang, South Sumatra, Indonesia; ^11^ School of Medicine Universitas Syiah Kuala, Dr. Zainoel Abidin Hospital, Banda Aceh, Aceh, Indonesia; ^12^ Faculty of Medicine Universitas Brawijaya, Saiful Anwar General Hospital, Malang, East Java, Indonesia; ^13^ Faculty of Medicine Universitas Sebelas Maret, dr. Moewardi General Hospital, Surakarta, Central Java, Indonesia; ^14^ Department of Internal Medicine, Primaya Hospital, Bekasi, West Java, Indonesia; ^15^ Faculty of Medicine Universitas Riau, Arifin Achmad General Hospital, Pekanbaru, Riau, Indonesia; ^16^ Faculty of Medicine Universitas Sam Ratulangi, Prof. dr. R. D. Kandou Hospital, Manado, North Sulawesi, Indonesia; ^17^ Faculty of Medicine Universitas Andalas, Dr. M. Djamil General Hospital, Padang, West Sumatra, Indonesia; ^18^ Department of Internal Medicine, Abdul Wahab Sjahranie Hospital, Samarinda, East Kalimantan, Indonesia; ^19^ Department of Internal Medicine, Indonesia Red Cross General Hospital, Bogor, West Java, Indonesia; ^20^ Faculty of Medicine Lampung University, Abdul Moelok Hospital, Bandar Lampung, Lampung, Indonesia; ^21^ Faculty of Medicine Universitas Lambung Mangkurat, Ulin Hospital, Banjarmasin, South Kalimantan, Indonesia; ^22^ Gunung Jati General Hospital, Cirebon, West Java 28 Emanuel Hospital, Banjarnegara, Central Java, Indonesia; ^23^ Faculty of Medicine Universitas Jenderal Soedirman, Dr. Margono Soekarjo Hospital, Purwokerto, Central Java, Indonesia; ^24^ Department of Internal Medicine, H. Abdurrahman Sayoeti General Hospital, Jambi, Riau, Indonesia; ^25^ Department of Internal Medicine, Kharitas Bhakti Hospital, Pontianak, West Kalimantan, Indonesia; ^26^ Department of Internal Medicine, Permata Hospital, Depok, West Java, Indonesia; ^27^ Department of Internal Medicine, Mayapada Hospital Jakarta Selatan, Jakarta, Special Capital Region of Jakarta, Indonesia; ^28^ Department of Internal Medicine, Awal Bros Hospital Batam, Riau Islands, Indonesia; ^29^ Faculty of Medicine Universitas Nusa Cendana, Wilhelmus Zakaria Johannes Hospital, Kupang, Indonesia; ^30^ Faculty of Medicine University of Mataram, West Nusa Tenggara Regional General Hospital, Mataram, West Nusa Tenggara, Indonesia; ^31^ Department of Internal Medicine, dr. Doris Sylvanus Hospital, Palangkaraya, Central Kalimantan, Indonesia; ^32^ Faculty of Medicine Alkhairaat University, Anutaparu General Hospital, Palu, Central Sulawesi, Indonesia; ^33^ Department of Internal Medicine, Jayapura Regional General Hospital, Jayapura, Papua, Indonesia; ^34^ Faculty of Medicine, Universitas Bangka Belitung, Pangkal Pinang, Bangka Belitung Islands, Indonesia; ^35^ Department of Internal Medicine, Harapan dan Doa Regional Hospital, Bengkulu, Bengkulu, Indonesia; ^36^ Department of Internal Medicine, Bahteramas General Hospital, Kendari, South East Sulawesi, Indonesia; ^37^ Department of Internal Medicine, Aloei Saboe Hospital, Gorontalo, Gorontalo, Indonesia; ^38^ Department of Internal Medicine, Piru Hospital, West Seram, Maluku, Indonesia; ^39^ Faculty of Medicine Universitas Negeri Khairun, Chasan Boesoirie General Hospital, Ternate, North Maluku, Indonesia; ^40^ Department of Internal Medicine, Sele be Solu Hospital, Sorong, West Papua, Indonesia

**Keywords:** obesity, internist, cardiovascular risk factor, occupational health, physicians

## Abstract

**Background:**

Obesity constitutes a significant global health concern, including in Indonesia, through increased risk of non-communicable diseases. Physicians, as healthcare providers, are not exempt from the impact of obesity toward general health, quality of life, and work performance. Among physicians, internists are particularly significant, as they assume primary responsibility for the management of obesity, thus the primary focus in this study. Obesity in internists is related to modifiable and non-modifiable risk factors. Therefore, identification of prevalence and risk factors of obesity in internists may aid in the improvement of their health through risk factor modification.

**Methods:**

A multicenter randomized cross-sectional study with a total sample of 1,064 internists across Indonesia is conducted to identify obesity profile and risk factors. Data were collected through questionnaire, physical examination, and biochemical testing and were analyzed using descriptive, bivariate, and multivariate analyses.

**Results:**

The prevalence of obesity in Indonesian internists is 61.4%, higher than the general population. Risk factors associated with obesity in Indonesian internists after adjusting for confounding factors were male gender (aOR 1.43, 95% CI 1.08-1.90), hypertension (aOR 1.88, 95% CI 1.26-2.79), history of diabetes mellitus (aOR 2.93, 95% CI 1.53-5.60), newly diagnosed diabetes mellitus (aOR 2.41, 95% CI 1.22-4.77), newly diagnosed prediabetes (aOR 1.70, 95% CI 1.26-2.30), and inadequate physical activity (aOR 1.85, 95% 1.15-2.98).

**Conclusion:**

Internists are a special population differing in prevalence of obesity and its related risk factors compared with the general population, due to high professional demand impacting healthy lifestyle and behavior.

## 1 Introduction

According to the World Health Organization (WHO), obesity is a global health challenge, with nearly 60% of the adult population living with overweight or obesity which has caused more than 1.2 million deaths each year through increased risk of non-communicable diseases (1, 2). In 2022, WHO estimated that 890 million adults lived with obesity (16% of population) (3). The prevalence of obesity has surged by 21% in the last decade (1), with developing countries experiencing a marked rise attributed to socioeconomic status and changes in habits (4, 5).

Reflecting this global trend, the latest 2018 national survey in Indonesia revealed that obesity prevalence in Indonesia has risen significantly, with 35.4% of obese adults, notably higher among women (44.4%) compared with men (26.6%) (6). Among healthcare workers, a systematic review from 83 studies across 29 countries conducted by Sadali et al. reported that obesity affected 16.3% nurses, underscoring the widespread impact of obesity within the healthcare workforce (7).

Physicians, as integral healthcare providers, are similarly impacted by obesity. Multiple cross-sectional studies have documented the prevalence of obesity among healthcare workers (including physicians) as follows: in 17% in the UK (8), 37.9% in Saudi Arabia (9), 28.4% in Kenya (10), 12.5%-28.9% in Ghana (11), 7.6% in Malaysia (12), 6.5% in Thailand (13), and 6.3% in Singapore (14). The presence of obesity elevates the risk of cardiometabolic diseases by increasing blood pressure, glycemic index, and dyslipidemia, while also impairing their occupational performance as healthcare providers (15, 16). Physicians with obesity and overweight were found to be less confident in educating patients about weight loss and healthy lifestyle and were more prone to distrust from the patients, and also patients were less likely to comply with their recommendations (16, 17).

Obesity among physicians is caused by modifiable or non-modifiable risk factors. Despite having better knowledge and health behavior compared with the general population, physicians often dismissed their personal health compared with occupational responsibility, which caused irregular working hours and dietary behavior and increased exposure to work-related stress (8, 18).

Considering the impact of obesity toward physicians, insights about the prevalence of obesity among physicians and its risk factors are needed to reduce the number of obesity through modification of risk factors, in order to increase their performance as healthcare providers. Among physicians, internists represent the most pertinent subgroup, as they bear primary responsibility for managing patients with chronic conditions, including obesity. Up until now, there is still no study on the prevalence of obesity and cardiometabolic risk factors among internists, although internists are physicians with close contact with patients of various ages and backgrounds and have the required competence to treat obesity and cardiometabolic diseases.

Therefore, this study is conducted to describe the profile of obesity among internists and cardiovascular risk factors influencing it. The results of this study serve to contribute to the improvement of internists’ health through modification of cardiovascular risk factors.

## 2 Methods

### 2.1 Study population and data retrieval

This is a cross-sectional study conducted in 39 branches of *Perhimpunan Dokter Spesialis Penyakit Dalam Indonesia* (PAPDI) from December 2023 to March 2024. The samples of this study are internists chosen randomly from a total of 5,436 members of PAPDI, allocating to 20% of members of each branch. The minimum sample size was calculated using a sample survey formula, because the population size, members of PAPDI, is known. The estimated proportion is 0.35 because the known obesity prevalence in Indonesia is 35.4%. The margin of error is determined as 0.03 (3%), and the Z-score for 95% confidence is 1.96. From this formula, a minimum sample size is set on 825 individuals. The stratified sampling approach was implemented to ensure that the sample accurately represented the diverse geographic distribution of internists across Indonesia. Random selection was performed using a random number generator available on the website random.org.

Data were collected through questionnaire taking, physical examination, and biochemistry laboratory examination. The questionnaire collects information on demographic characteristics, health profiles, cardiometabolic risk factors, and health behavior. Physical examinations consisted of anthropometric and blood pressure measurements, followed by lab examinations of fasting glucose and HbA1c levels. All physical examinations were conducted by trained general practitioner, and blood samples were analyzed at a nationally accredited laboratory.

### 2.2 Definitions

The definition of obesity in this study is the Asia Pacific WHO Obesity Criteria, which constitutes Class II obesity (BMI ≥30 kg/m^2^), class I obesity (BMI 25-29.9 kg/m^2^), at risk (BMI 23-24.9 kg/m^2^), normal (BMI 18.5-22.9 kg/m^2^), underweight (BMI <18.5 kg/m^2^) (19). Gender, age, and marital status were in accordance with identity card. Diabetes mellitus is defined as fasting glucose level of ≥126 mg/dL or HbA1c ≥6.5%. Prediabetes is characterized by a fasting plasma glucose level ranging from 100 to 125 mg/dL or an HbA1c level between 5.7% and 6.4% (20). Hypertension and dyslipidemia in this study are defined as having been diagnosed by physicians or were consuming antihypertensive or dyslipidemia medications. Physical activity is considered adequate if the WHO criteria is fulfilled, which is moderate intensity physical activity at least 150 min/week or high intensity at least 75 min/week (or combination of both), plus muscle resistance training at least twice a week. Residence locations were categorized as urban or rural according to the village category by *Badan Pusat Statistik* (BPS) (21). Smoking was classified into active smoker, ex-smoker, and non-smoker.

### 2.3 Statistical analysis

Data of this research were described in categorical data and were analyzed through three steps, which were descriptive analysis, bivariate analysis, and multivariate analyses. The BMI variable is categorized by the Asia Pacific WHO Obesity Criteria; furthermore, for bivariate analysis, it is categorized into dichotomous variable, which are obese (Class I obesity and Class II obesity) and not obese (underweight, normal, and at risk). Descriptive data are shown in proportion; bivariate analysis was conducted using chi square for categoric variables to gain the odds ratio with a confidence interval of 95% and a p value of 0.05. Variables with a p value of <0.2 are included into the logistic regression model for multivariate analysis, to achieve the adjusted odds ratio with 95% confidence interval. Variables that show a p value of <0.05 are deemed significant variables. Hosmer–Lemeshow goodness-of-fit test shows that the model’s predictions are a good match for the actual data. A p-value of >0.05 indicates a good fit for the logistic regression model. The Nagelkerke R^2^ value indicates the power of explanation of the logistic regression model, with a higher value indicating a better model fit (22).

### 2.4 Ethics statement

Ethical approval for the study was obtained from the Ethical Committee Faculty of Medicine University of Indonesia. Informed consent was waived at the beginning of each questionnaire form. Access to the data is restricted to the research team, and participant identities are kept confidential. Participants who declined to participate in this study and have not completed all the research data will not be included in the study analysis.

## 3 Results

### 3.1 Clinical characteristics

A total of 1,064 subjects (67.8% response rate) completed all steps of the study (questionnaire, physical examination, biochemical examination), out of 1,568 samples selected randomly from 5,436 internists in Indonesia. Baseline characteristics of the patients are shown in **Table 1**. The distributions of risk factor assessed in this study are shown in **Table 2**.

**Table 1:** Baseline characteristics of sample.

Parameter	n (%)
Gender	
Male	612 (57.5)
Female	452 (42.5)
Age	
25–34 yo	139 (13.1)
35–49 yo	600 (56.4)
50–64 yo	264 (24.8)
≥65 yo	61 (5.7)
Obesity	
Underweight	13 (1.2)
Normal	184 (17.3)
At risk	214 (20.1)
Obese I	440 (41.4)
Obese II	213 (20.0)
Central obesity	760 (71.4)
Hypertension	
Hypertension	198 (18.6)
Without hypertension	866 (81.4)
Dyslipidemia	
Dyslipidemia	352 (33.1)
No dyslipidemia	712 (66.9)
Diabetes mellitus	
Had history of diabetes mellitus	80 (7.5)
Newly diagnosed diabetes mellitus	51 (4.8)
Newly diagnosed prediabetes	338 (31.8)
Without diabetes	595 (55.9)
Family history of early premature atherosclerosis	
Yes	147 (13.8)
No	917 (86.2)
Residence location	
Urban	987 (92.8)
Rural	77 (7.2)
Marital status	
Single	79 (7.4)
Married	943 (88.6)
Widow	42 (3.9)
Smoking	
Smoker	30 (2.8)
Ex-smoker	89 (8.4)
Non-smoker	945 (88.8)
Physical activity	
Adequate	82 (7.7)
Inadequate	982 (92.3)
Sleep duration	
<7 h/day	670 (63)
7-8 h/day	391 (36.7)
≥9 h/day	3 (0.3)
Weekly working hours	
<55 h	506 (47.6)
≥55 h	558 (52.4)

**Table 2:** Descriptive statistics of risk factors.

Parameter	Not obese (N = 197)	At risk (N = 214)	Obese I (N = 440)	Obese II (N = 213)
Gender				
Male, *n (%)*	78 (12.8)	120(19.6)	266 (43.5)	148 (24.2)
Female, *n (%)*	119(26.3)	94(20.8)	174 (38.5)	65 (14.4)
Age				
25–34 years old, *n (%)*	19(13.6)	36(2.9)	55(39.6)	29(20.9)
35–49 years old, *n (%)*	120 (20)	106(17.7)	260(43.3)	114(19)
50–64 years old, *n (%)*	45(17.1)	57(21.6)	102(38.6)	60(22.7)
≥65 years old, *n (%)*	13 (21.3)	15(24.6)	23 (37.7)	10(16.4)
Hypertension				
Hypertension, *n (%)*	13(6.6)	29 (14.6)	85 (42.9)	71 (35.9)
Without hypertension, *n (%)*	184(21.3)	185(21.4)	355 (41)	142 (16.4)
Dyslipidemia				
Dyslipidemia, *n (%)*	36(10.3)	68(19.3)	160 (45.5)	88 (25)
No dyslipidemia, *n (%)*	161 (22.6)	146(20.5)	280 (39.3)	125 (17.6)
Diabetes mellitus				
Had history of diabetes Mellitus, *n (%)*	8(10)	5(6.3)	36 (45)	31 (38.8)
Newly diagnosed Diabetes mellitus, *n (%)*	2(3.9)	10 (19.6)	25 (49)	14 (27.5)
Newly diagnosed Prediabetes, *n (%)*	33 (9.8)	68(20.1)	146(43.2)	91(26.9)
Without diabetes, *n (%)*	154(25.9)	131(22)	233(39.2)	77(12.9)
Family history of early premature atherosclerosis				
Yes, *n (%)*	21 (14.3)	24(16.3)	66 (44.9)	36 (24.5)
No, *n (%)*	176 (19.2)	190 (20.7)	374 (40.8)	177 (19.3)
Location of residence				
Urban, *n (%)*	184(18.6)	199(20.2)	404 (40.9)	200 (20.3)
Rural, *n (%)*	13 (16.9)	15(19.5)	36 (46.8)	13 (16.9)
Marital status				
Single, *n (%)*	14 (17.7)	18(22.8)	31 (39.2)	16 (20.3)
Widow, *n (%)*	7 (16.7)	9 (21.4)	16 (38.1)	10 (23.8)
Married, *n (%)*	176(18.7)	187 (19.8)	393 (41.7)	187 (19.8)
Smoking				
Smoker, *n (%)*	4 (13.3)	4(13.3)	12 (40.0)	10 (33.3)
Ex-smoker, *n (%)*	9(10.1)	16(18)	41 (46.1)	23 (25.8)
Non-smoker, *n (%)*	184(19.5)	194(20.5)	387 (41)	180 (19)
Physical activity				
Inadequate, *n (%)*	186(18.9)	184 (18.7)	412 (42)	200 (20.4)
Adequate, *n (%)*	11 (13.4)	30(36.6)	28 (34.1)	13 (15.9)
Sleep duration				
<7 h/day, *n (%)*	126(18.8)	131(19.6)	268 (40)	145 (21.6)
7-8 h/day, *n (%)*	71 (18.1)	82 (21)	171 (43.7)	67 (17.1)
≥9 h/day, *n (%)*	0 (0)	1 (33.3)	1 (33.3)	1 (33.3)
Working hours				
≥55 h/week	35 (19.7)	35 (19.7)	239(43.8)	121 (16.9)
<55 h/week	32 (17.9)	37(20.7)	201 (39.1)	92 (22.3)

**Figure 1:**
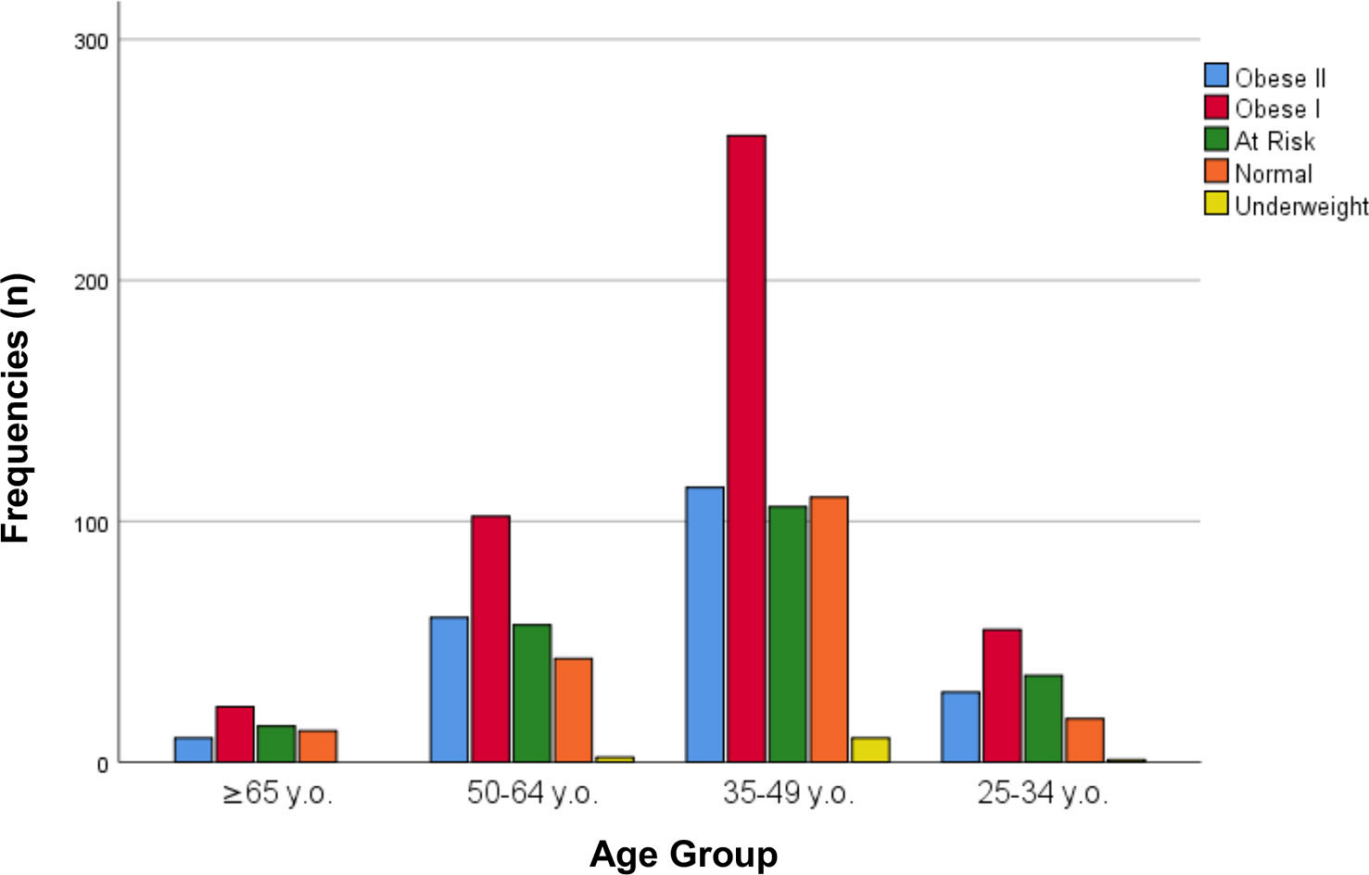
Obesity in internists based on age group.

**Figure 2:**
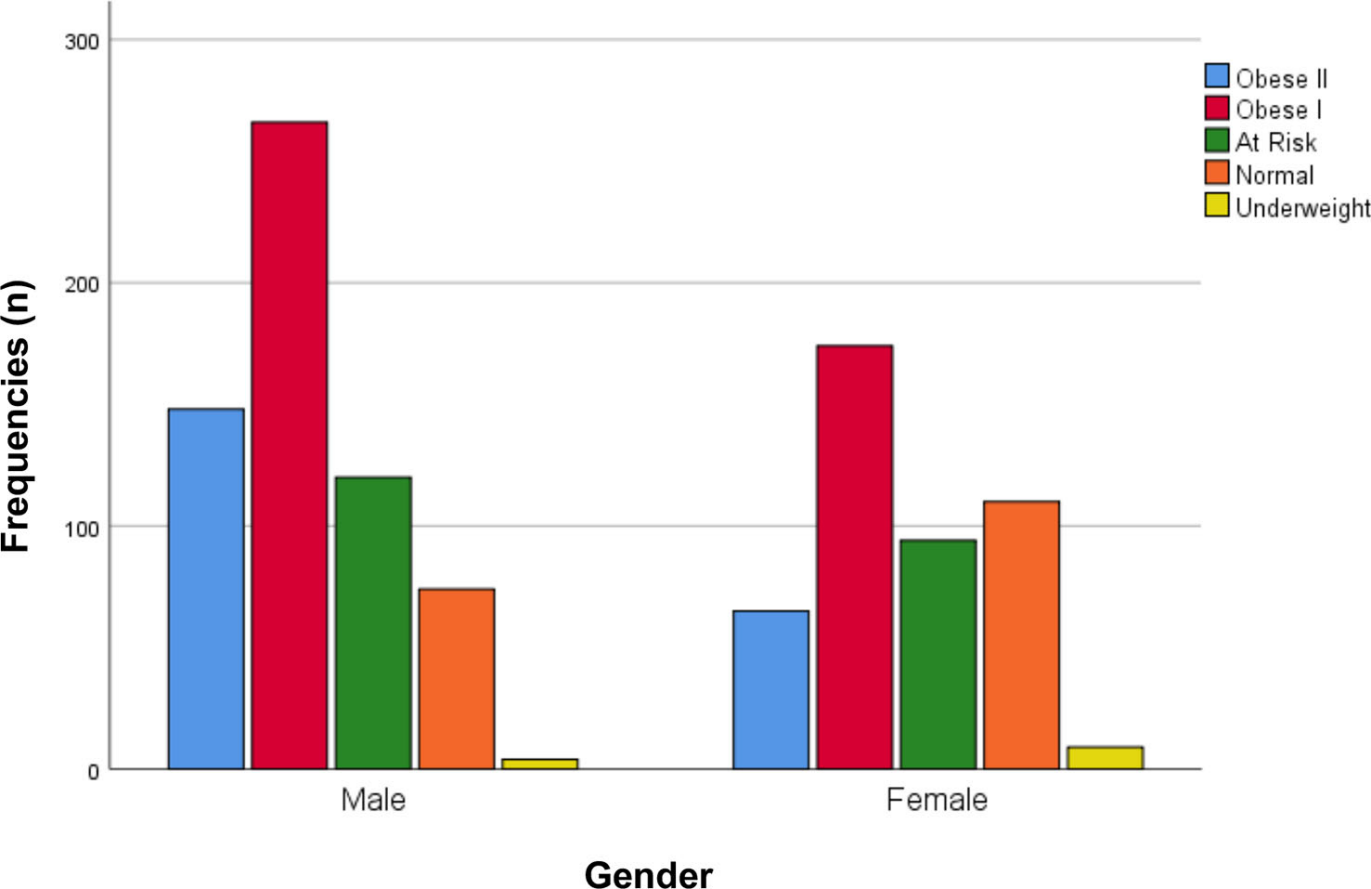
Obesity in internists based on gender.

**Figure 3:**
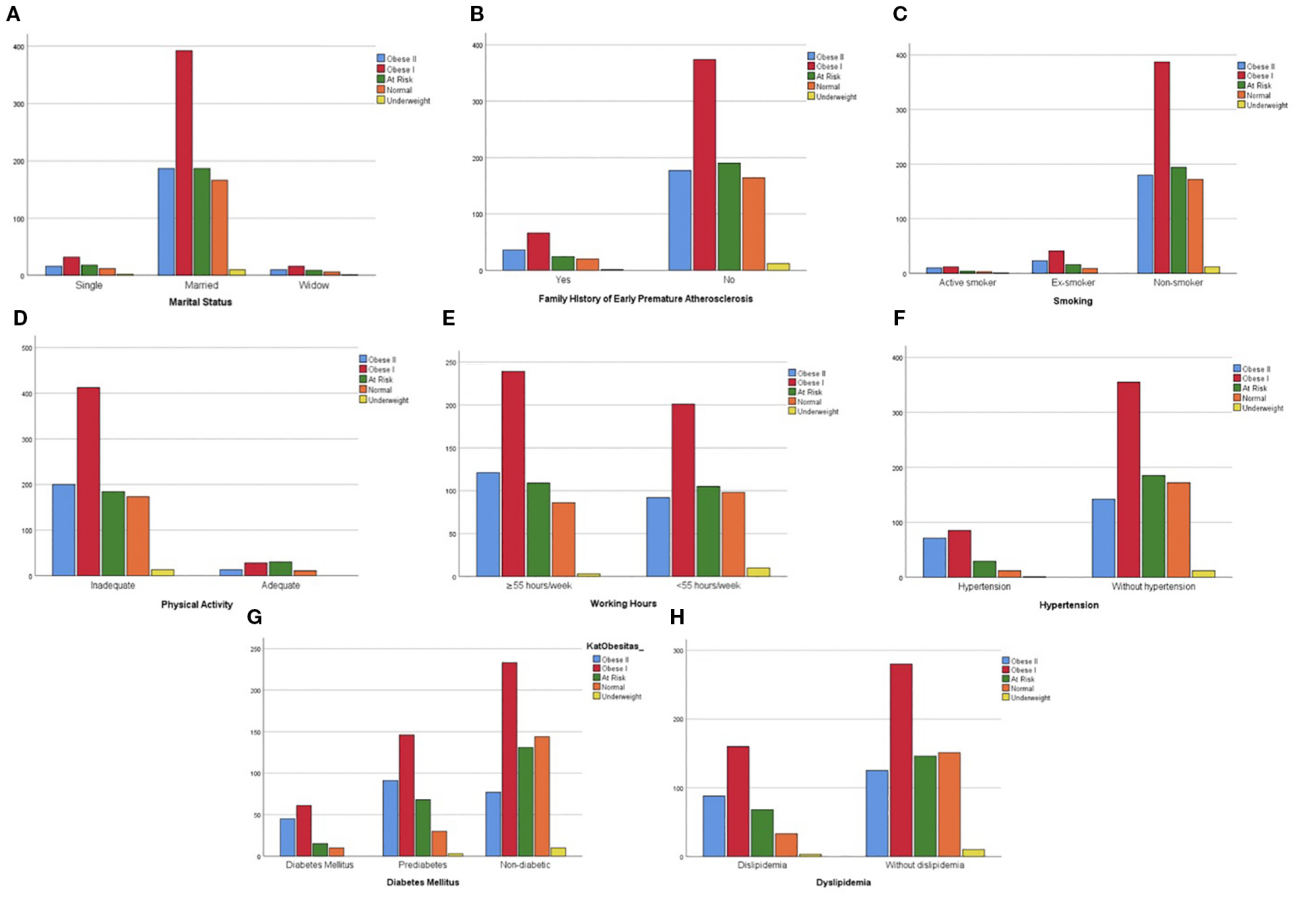
Obesity in internists based on risk factors: **(A)** marital status, **(B)** family history of early premature atherosclerosis, **(C)** smoking, **(D)** physical activity, **(E)** working hours, **(F)** hypertension, **(G)** diabetes mellitus, **(H)** dyslipidemia.

Obesity was found in 653 (61.4%) internists in this study based on the WHO Asia Pacific criteria (41.4% obese I, 20.0% obese II). Based on age distribution, 600 (56.4%) internists with obesity were 35–49 years old, 264 (24.8%) 50–64 years old, 139 (13.1%) 25–34 years old, and 61 (5.7)% aged ≥65 years old (**Figure 1**). Out of 653 obese samples, 414 (63.4%) were men and 239 (36.6%) were women (**Figure 2**). Looking on the comorbidities, 156 (23.9%) internists with obesity had hypertension, 248 (38.0%) had dyslipidemia, 106 (16.2%) had diabetes mellitus, and 102 (15.6%) had families with early premature atherosclerosis. Majority of obese internists were married (580 or 88.9%). Lastly, 86 (13.1%) internists with obesity smoke (current or former smokers), 612 (93.7%) did not meet the required physical activity level according to WHO, 413 (63.2%) slept less than 7 h/day, and 360 (55.1%) worked ≥55 h/week (**Figure 3**). Out of all variables, the strongest predictors of obesity was physical inactivity.

### 3.2 Bivariate analysis of obesity risk factors

Based on bivariate analysis, as shown in **Table 3**, risk factors that correlated significantly with obesity in internists were men (OR 1.86, 95% CI 1.45-2.39, p<0.001), hypertension (OR 2.76, 95% CI 1.91-3.98, p<0.001), dyslipidemia (OR 1.81, 95% CI 1.38-2.38, p<0.001), had history of diabetes mellitus (OR 4.74, 95% CI 2.56-8.77, p<0.001), newly diagnosed diabetes mellitus (OR 2.99, 95% CI 1.53-5.82, p=0.001), newly diagnosed prediabetes (OR 2.16, 95% CI 1.63-2.86, p<0.001), family history of early premature atherosclerosis (OR 1.51, 95% CI 1.04-2.19, p=0.032), ex-smoker (OR 1.71, 95% CI 1.06-2.76, p=0.029), inadequate physical activity (OR 1.65, 95% 1.05-2.60, p=0.028), and working hour ≥55 h/week (OR 1.32, 95% CI 1.03-1.69, p=0.027).

**Table 3:** Associations between cardiovascular risk factors and obesity in internists.

Risk factors	Obese n (%) (N = 653)	Not obese n (%) (N = 411)	Bivariate		Multivariate	
			OR (95% CI)	P-value	aOR (95% CI)^*^	P-value
Gender						
Male	414 (63.4)	198 (48.2)	**1.86 (1.45-2.39)**	**0.000**	**1.43 (1.08-1.90)**	**0.013**
Female	239 (36.6)	213 (51.8)				
Age						
25–34 years old	84(12.8)	55(13.4)	Ref			
35–49 years old	374 (57.3)	226 (55.0)	1.08(0.74-1.58)	0.677	–	–
50–64 years old	162 (24.8)	102 (24.8)	1.04(0.68-1.58)	0.855		
≥65 years old	33 (5.1)	28 (6.8)	0.77(0.42-1.42)	0.403		
Hypertension						
Hypertension	156 (23.9)	42 (10.2)	**2.76 (1.91-3.98)**	**0.000**	**1.88 (1.26-2.79)**	**0.002**
Without hypertension	497 (76.1)	369 (89.8)				
Dyslipidemia						
Dyslipidemia	248 (38.0)	104 (25.3)	**1.81 (1.38-2.38)**	**0.000**	1.302 (0.97-1.75)	0.079
Without dyslipidemia	405 (62.0)	307 (74.7)				
Diabetes mellitus						
Had history of diabetes mellitus	67(10.3)	13(3.2)	**4.74(2.56-8.77)**	**0.000**	**2.93(1.53-5.60)**	**0.001**
Newly diagnosed diabetes mellitus	39 (6.0)	12 (2.9)	**2.99 (1.53-5.82)**	**0.001**	**2.41 (1.22-4.77)**	**0.012**
Newly diagnosed prediabetes	237(36.2)	101(24.6)	**2.16(1.63-2.86)**	**0.000**	**1.70(1.26-2.30)**	**0.001**
Without Diabetes	310 (47.5)	285 (69.3)	Ref			
Family history of early premature atherosclerosis						
Yes	102 (15.6)	45 (10.9)	**1.51 (1.04-2.19)**	**0.032**	1.26 (0.85-1.87)	0.252
No	551 (84.4)	366 (89.1)				
Location of residence						
Urban	604 (92.5)	383 (93.2)	0.901 (0.557-1.459)	0.672	–	–
Rural	49 (7.5)	28 (6.8)				
Marital status						
Single/widow	580 (88.8)	363 (88.3)	1.05 (0.71-1.55)	0.803	–	–
Married	73 (11.2)	48 (11.7)				
Smoking						
Smoker	22 (3.4)	8 (1.9)	1.83 (0.81-4.16)	0.147	1.37 (0.59-3.22)	0.466
Ex-smoker	64(9.8)	25(6.1)	**1.71(1.06-2.76)**	**0.029**	1.08(0.64-1.82)	0.771
Non-smoker	567 (86.8)	378 (92.0)	Ref			
Physical Activity						
Inadequate	612 (93.7)	370 (90.0)	**1.65 (1.05-2.60)**	**0.028**	**1.85 (1.15-2.98)**	**0.012**
Adequate	41 (6.3)	41 (10.0)				
Duration of sleep						
<7 h/day	413 (63.2)	257 (62.5)	1.03 (0.80-1.33)	0.814	–	–
>7 h/day	240(36.8)	154(37.5)				
Working hours						
≥55 h/week	360 (55.1)	198 (48.2)	**1.32 (1.03-1.69)**	**0.027**	1.20 (0.92-1.56)	0.175
<55 h/week	293 (44.9)	213 (51.8)				

^*^aOR, adjusted odds ratio, adjusted for risk factors with multivariate p value <0.2: gender, age, hypertension, dyslipidemia, diabetes mellitus, family history of early premature atherosclerosis, location of practice, smoking, physical activity, and working hours. *bold values indicates significant p value (p< 0.05), therefore are included in multivariate analysis.

### 3.3 Multivariate analysis of obesity risk factors

All significant variables from the bivariate analysis were included in the logistic regression model for multivariate analysis. Other variable that still showed a p value of <0.2 was also included in the model, which was smoker (p=0.147). The Hosmer and Lemeshow goodness-of-fit test showed a good fit with a p value of 0.312. Nagelkerke R^2^ showed a weak-to-moderate relationship between predictors and the outcome, with a value of 0.114.

After adjusting for confounding risk factors, as shown in **Table 3**, multivariate analysis showed that obesity in internists was significantly associated with male gender (aOR 1.43, 95% CI 1.08-1.90, p=0.013), hypertension (aOR 1.88, 95% CI 1.26-2.79, p=0.002), had history of diabetes mellitus (aOR 2.93, 95% CI 1.53-5.60, p=0.001), newly diagnosed diabetes mellitus (aOR 2.41, 95% CI 1.22-4.77, p=0.012), newly diagnosed prediabetes (aOR 1.70, 95% CI 1.26-2.30, p=0.001), and inadequate physical activity (aOR 1.85, 95% 1.15-2.98, p=0.012).

## 4 Discussion

Obesity is a prevalent health problem among internists in Indonesia. Prevalences of obesity in Indonesian internists are 61.4% based on the Asia Pacific WHO criteria (BMI>25) and 20.0% based on the Global WHO Obesity criteria (IMT>30), bigger than the prevalence of obesity (BMI>25) in the general adult population aged >18 years old in Indonesia (35.4%) (6). Our neighbor country, Malaysia, had also conducted a similar study, reporting an obesity prevalence of 21.1% among healthcare workers, with 7.6% of them being physicians. There were no specific reported numbers among physicians. The prevalence might be lower because the obesity criterion used was BMI >30, whereas this study used a stricter cutoff value of BMI >25 from Asia Pacific WHO (12).

This study found an increased risk of obesity in male internists (OR 1.86, 95% CI 1.45-2.39; aOR 1.43, 95% CI 1.08-1.90), conflicting the previous studies’ results which had found an increased risk in women due to biological and psychosocial factors affecting dietary behavior (12, 23). However, accounting only healthcare workers, there were studies stating an increased risk of obesity in men compared with women. This was elaborated by occupational risk factors playing a big role in obesity in healthcare workers besides biological factors. In Indonesia, the regulation states that each doctor may practice medicine in up to three different healthcare facilities. Therefore, due to sociocultural factors in Southeast Asia that predominantly position men as the primary providers for their families, male internists in Indonesia may work longer hours, which consequently increases their risk of obesity. In assessing gender-specific risk factors of obesity, healthcare workers had the highest prevalence of obesity in men, whereas in women the occupation with the highest prevalence of obesity was agriculture. Without adjustments of stress and comorbidities, men who worked in shifts were also found to have increased risk of obesity (24).

History of smoking (ex-smokers) increased the risk of obesity in Indonesian internists (OR 1.71, 95% CI 1.06-2.76), although its significance disappeared when adjusted for confounding factors. Smoking was believed to have a role in weight loss and reducing appetite (25, 26). However, there are still obese populations in active smokers, and active smokers were found to increase the risk of central obesity compared with non-smokers in overweight or obese populations, especially in women, heavy smokers, and ex-smokers (27). Central obesity in heavy smokers was caused by the preponderance of also unhealthy lifestyle in heavy smokers, including alcohol consumption, sedentary lifestyle, and the changes of the HPA axis in patients with underlying obesity (27). Meanwhile, smoking cessation caused obesity through the withdrawal of nicotine effect on ghrelin and appetite (26–28). A meta-analysis found that smoking cessation could increase the mean body weight by 4.1 kg compared with active smokers of only 1.5 kg in 5.2 years (29). This increase is felt especially in the first 3 months of smoking cessation with risk predictors of underlying obesity, age less than 55 years old, and heavy smokers of 25 cigarettes/day (26).

Even though some studies stated that more physicians and medical students fulfilled the required physical activity compared with the general population (30, 31), this study found that majority of internists in Indonesia (92.3%) did not fulfill the required physical activity recommended by WHO. Inadequate physical activity increased the risk of obesity in Indonesian internists (OR 1.65, 95% CI 1.05-2.60; aOR 1.85, 95% CI 1.15-2.98), as theoretically physical activity could reduce adipose tissues involved in low-grade chronic inflammation in obesity (32).

Working hours of ≥55 h/week is also associated with obesity in internists (OR 1.32, 95% CI 1.03-1.69), although this association is insignificant when controlled for confounders. This association could be elaborated through the impact of high working hours with the lifestyle of the physician; as found in a study, overtime work (more than 65 h/week) in physicians is associated with less physical activity, skipping breakfast, and sleeping less than 6 h/day (33). Increased risk of obesity in long working hours was also found in multiple studies, especially in women (34–36).

Hypertension and diabetes mellitus significantly increased the risk of obesity in internists even after controlling for confounders. The thickness of pericardial adipose tissue was stated to play a role in insulin resistance and increase in blood pressure (15). Premature early atherosclerosis in family also increased the risk of obesity in internists, although this association is insignificant when controlled for confounders (OR 1.51, 95% CI 1.04-2.19; aOR 1.26, 95% CI 0.8-1.87). History of cardiovascular and metabolic diseases was related to the increase of childhood onset obesity and the severity of obesity (37). In contrast, obesity related to dyslipidemia, insulin resistance, and endothelial dysfunction also played a role in atherosclerosis and the development of atherosclerotic heart diseases (15). Obesity could cause coronary heart diseases through hypertension, dyslipidemia, and diabetes, although obesity alone was still found to cause coronary heart diseases without those risk factors and comorbidities (15).

Results from this study may serve as a foundation to provide better insight on managing the obesity epidemic in Indonesia, which apparently is also prevalent among Indonesian internists. As the main physicians that manage obesity and other metabolic diseases, internists shall provide a better example of healthy lifestyle for their respective patients (17). Known risk factors of obesity from this study, which are male, hypertension, diabetes mellitus, and inadequate physical activity, should be addressed by PAPDI, as the governing organization of Indonesian internists. Therefore, results from this study may support institutional policy changes, such as providing facilities to increase physical activity in the workday and special occasions during the national event gatherings.

### 4.1 Limitations

This is the first study in Indonesia that tried to discover the obesity epidemic among Indonesian physicians. However, there were limitations in the conduct of this study. The study design used was cross-sectional; therefore, the variables found to increase the risk of obesity could not be directly inferred as the cause of obesity. Additionally, the Nagelkerke R² value of 0.114 indicates that the predictors in the model explain only a small portion of the variability in obesity risk, suggesting that other important factors not included in the model may influence the outcome. Another limitation in this study was the data collection of metabolic risk factors, which were self-reported by the participant; therefore, there might be some recall bias. Future research should consider longitudinal study designs to better establish causal relationships and include more comprehensive and objective measurements of metabolic and behavioral factors to improve model predictability and better understand the multifactorial nature of obesity in this population.

## 5 Conclusion

Obesity is still a burdening health problem among internists as healthcare providers. The prevalence of obesity among Indonesian internists is higher than the general population and was found to be related to male, hypertension, diabetes mellitus, prediabetes, and inadequate physical activity. Findings of associated risk factors for obesity in internists may serves as a basis to improve the health of internists.

## Data Availability

The original contributions presented in the study are included in the article/supplementary material. Further inquiries can be directed to the corresponding author.
